# Photosynthetic performance of spring maize leaves and the development and formation of superior and inferior grains

**DOI:** 10.3389/fpls.2025.1732666

**Published:** 2026-01-09

**Authors:** Wenzhuo Cao, Zhenwen Yu, Yu Shi, Zhen Zhang, Yongli Zhang

**Affiliations:** 1 National Key Laboratory of Wheat Breeding, Agricultural College, Shandong Agricultural University, Tai’an, China; 2 Key Laboratory of Crop Physiology, Ecology and Tillage, Ministry of Agriculture and Rural Affairs, Shandong Agricultural University, Tai’an, China

**Keywords:** endogenous hormones, enzyme activity, nitrogen fertilization rate, starch accumulation dynamics, yield

## Abstract

**Introduction:**

Nitrogen application is crucial for enhancing maize yield and optimizing grain filling. However, the effects of nitrogen fertilization on post–anthesis leaf photosynthetic performance, starch accumulation dynamics in superior and inferior grains, grain development, and yield remain unclear.

**Methods:**

A field experiment with five nitrogen fertilization rates (0 [N0], 90 [N90], 135 [N135], 180 [N180], and 225 [N225] kg ha^–1^) was conducted in the black soil region of Northeast China during the spring maize growing seasons of 2022 to 2024.

**Results:**

Compared with the other treatments (N0, N90, N135, and N225), the N180 treatment significantly increased the activities of superoxide dismutase (SOD) and peroxidase (POD) in ear leaves after anthesis, optimized the balance between zeatin riboside (ZR) and abscisic acid (ABA), delayed leaf senescence, and thereby increased the net photosynthetic rate by 1.8–76.6%. Meanwhile, compared to the other treatments, the N180 treatment enhanced the activities of ADP–glucose pyrophosphorylase (AGPase) and soluble starch synthase (SSS) in both superior and inferior grains by 7.7–49.3% and 7.4–36.9%, respectively, compared with the N0, N90, N135, and N225 treatments. This improvement optimized the endogenous hormone balance in grains, increased starch accumulation rates in both superior and inferior grains, prolonged the active starch accumulation period, and promoted grain filling. The synergistic improvement in source leaf photosynthetic performance and grain sink activity ultimately enabled the N180 treatment to coordinately increase the kernel number per ear and 100–kernel weight, resulting in a yield increase of 5.8–55.7% compared with the N0, N90, N135, and N225 treatments.

**Discussion:**

Future studies may further reveal the physiological and molecular mechanisms by which nitrogen coordinates source–sink functions from perspectives such as hormonal signaling and the regulation of key enzyme gene expression.

## 1 Introduction

The black soil region of Northeast China is one of the world’s four major black soil zones, accounting for 31.2% of the total maize production in China (Qian et al., 2025). Achieving and sustaining high maize yields in this region relies on the management of key nutrients such as nitrogen. Leaf photosynthesis serves as the material foundation for maize yield formation (Wang et al., 2024c). However, gradual leaf senescence after anthesis leads to a decline in photosynthetic capacity and a reduced supply of assimilates, which becomes a key factor limiting grain filling and yield improvement (Du et al., 2023). The process of leaf senescence is jointly regulated by reactive oxygen species (ROS) scavenging mechanisms [involving enzymes such as superoxide dismutase (SOD) and peroxidase (POD)] and the balance of endogenous hormones [particularly the antagonistic interaction between zeatin riboside (ZR) and abscisic acid (ABA)] (Huang et al., 2022). Appropriate nitrogen application can maintain the ultrastructural integrity of chloroplasts and increase chlorophyll content (Liu et al., 2018), thereby delaying post–anthesis leaf senescence and enhancing light energy absorption and conversion efficiency (Cheng et al., 2025). Furthermore, nitrogen can promote root growth and energy (ATP) metabolism, improving the uptake and utilization efficiency of other nutrients such as phosphorus, which in turn fosters the formation and translocation of photosynthetic products (Rebi et al., 2022). Therefore, optimizing nitrogen application to delay leaf senescence and enhance photosynthetic performance is a crucial approach for ensuring high maize yield and sustainable production.

Research findings indicate that maize yield is directly determined by the filling degree of grain sink capacity. Starch, a major component of grains, constitutes approximately 70% of the grain dry weight, and its synthesis and accumulation form the basis for grain filling and yield formation (Jiang et al., 2025). Due to differences in kernel positions on the maize ear, kernels located in the middle and lower parts with faster filling rates are referred to as superior grains, whereas those at the top with delayed filling are termed inferior grains (Liu et al., 2020). Studies have shown that compared to inferior grains, superior grains exhibit significantly higher average and maximum grain–filling rates, resulting in higher kernel weight. The imbalance in grain filling between superior and inferior grains has become a key factor limiting maize yield improvement (Yu et al., 2024). Appropriate nitrogen fertilization rates facilitate the development and filling of inferior grains in maize, reducing the grain abortion rate. Conversely, insufficient or excessive nitrogen application intensifies competition for photoassimilates between superior and inferior grains, leading to impaired filling and reduced kernel weight in inferior grains (Shen et al., 2017). Research on wheat has indicated that nitrogen application rates have a more pronounced effect on the grain–filling characteristics of inferior grains (Liang et al., 2017). Optimal nitrogen management can shorten the early grain–filling phase and prolong the mid–to–late filling phase, and increase the average filling rate of inferior grains, thereby reducing kernel weight differences within the ear and enhancing overall grain weight (Wang et al., 2024a). These studies suggest that optimizing nitrogen application is beneficial for improving the grain–filling balance between superior and inferior grains and promoting the coordinated filling of grain sink capacity. However, research on the starch accumulation characteristics of spring maize superior and inferior grains and their response to nitrogen remains relatively limited, and the underlying physiological regulatory mechanisms are still unclear.

ADP–glucose pyrophosphorylase (AGPase) is a rate–limiting enzyme that catalyzes the initial reaction of starch synthesis, and its activity is significantly positively correlated with starch accumulation (V et al., 2019). Soluble starch synthase (SSS) is a key enzyme that catalyzes amylopectin chain elongation. High SSS activity can indirectly maintain high AGPase activity by reducing the feedback inhibition of AGPase through ADP–glucose accumulation (Liu et al., 2021), thereby promoting starch synthesis and accumulation. Studies have shown that with increasing nitrogen application rates, the activities of AGPase and SSS initially increase and then decrease. Under an optimal nitrogen application rate (200 kg ha^–1^), both activities reach their highest levels, leading to a 4.4–7.3% increase in total starch content (Wang et al., 2024a). Additionally, appropriate nitrogen application can significantly promote grain filling by regulating endogenous hormone levels in grains (Liu et al., 2021). Among the major endogenous hormones, auxin (IAA) promotes endosperm cell division and growth, while gibberellin (GA) increases endosperm cell volume (Gao et al., 2018; Yue et al., 2022a). Elevated IAA and GA contents, particularly in inferior grains under higher nitrogen levels (360 kg ha^–1^), are beneficial for enhancing grain sink capacity and sink strength (Wei et al., 2019). Abscisic acid (ABA) maintains sink activity during the mid–to–late filling stages by promoting the expression of AGPase and SSS genes and increasing enzyme activity (Yang et al., 2019), directly improving starch synthesis efficiency. However, excessively high ABA levels may be detrimental to grain filling. Research indicates that excessive nitrogen application (300 kg ha^–1^) can lead to excessively high ABA content in grains during the late filling stage, disrupting the balance among ABA, IAA, and zeatin + zeatin riboside (Z+ZR) and thereby inhibiting grain filling. Moreover, the peak contents of IAA and ABA are significantly positively correlated with the average and maximum grain–filling rates (Li et al., 2019). Based on this, we hypothesize that the effect of nitrogen application rate on starch accumulation and sink filling in superior and inferior grains may be mediated through the regulation of key starch–synthesizing enzyme activities and the dynamic balance of endogenous hormones.

Maize yield is closely related to leaf photosynthetic performance and the dynamic characteristics of starch accumulation in superior and inferior grains. Enhancing the leaf photosynthetic capacity and promoting effective starch accumulation in superior and inferior grains are important pathways for increasing maize yield (Liu et al., 2024). However, we still lack a systematic understanding of how nitrogen application rates, during the critical post–anthesis grain–formation stage, regulate endogenous hormone levels and key enzyme activities to achieve “source–sink” coordination and promote the filling of sink capacity in superior and inferior grains. To address this, we conducted a three–year field experiment in the main maize–production region of Northeast China. The objectives of this study were: (1) to clarify the dynamic changes in photosynthetic characteristics and senescence of maize leaves after anthesis under different nitrogen application rates; (2) to elucidate the effects of different nitrogen application rates on the dynamic characteristics of starch accumulation in superior and inferior grains of maize; and (3) to reveal the physiological mechanisms by which nitrogen application rates regulate the balance of endogenous hormones and the activities of key starch–synthesizing enzymes, thereby influencing starch synthesis and sink filling in superior and inferior grains.

## 2 Materials and methods

### 2.1 Test materials and design

The experiment was conducted in the spring maize growing seasons of 2022, 2023, and 2024 at the experimental field of Jilin Academy of Agricultural Sciences (43°29′55″N, 124°48′43″E), located in Gongzhuling City, Jilin Province. The region has a temperate continental monsoon climate, with an average annual temperature of 5.6°C and an average annual precipitation of 594.8 mm. The soil of the experimental site is medium–thick, typical black soil. The average soil nutrient contents in the 0–20 cm tillage layer before sowing in 2022, 2023 and 2024 were as follows: organic matter, 27.8 g kg^–1^; total nitrogen, 1.56 g kg^–1^; quick–acting phosphorus, 99.9 mg kg^–1^; and quick–acting potassium, 166.1 mg kg^–1^. The maize variety for the experiment was ‘Fumin 985.’ Five nitrogen (N) fertilization rates were established: 0 (N0), 90 (N90), 135 (N135), 180 (N180), and 225 (N225) kg ha^–1^. Nitrogen fertilizer was split–applied, with 40% as basal fertilizer before sowing and 60% at the jointing stage. Phosphorus (P_2_O_5_ at 75 kg ha^–1^) and potassium (K_2_O at 90 kg ha^–1^) fertilizers were both applied basally before sowing. The N, P, and K fertilizers were applied in the form of urea, calcium superphosphate, and potassium chloride, respectively. A randomized complete block design with three replications was adopted. Each plot area was 200 m^2^ (20 ×10 m), with a planting density of 60,000 plants ha^–1^. Sowing dates were May 12, 2022; May 1, 2023; and April 27, 2024, with corresponding harvest dates on October 6, 2022; September 26, 2023; and September 25, 2024. Post–harvest, straw was returned to the field, followed by deep loosening and deep plowing. All other field management practices followed local high–yield production standards.

### 2.2 Determination items and methods

#### 2.2.1 Grain yield and yield components

At maturity, three rows of maize were harvested from the center of each plot and the number of effective ears was counted (this procedure was repeated three times). From each replicate, 30 representative ears were randomly selected and air–dried, and the number of kernels per ear and the 100–kernel weight was determined. Grain yield was calculated based on a moisture content of 14%.

#### 2.2.2 Grain hormone content

At anthesis, uniformly growing plants were selected and tagged. Every 12 days from 12 to 60 days after anthesis, three representative ears were collected from the tagged plants between 9:00 and 10:00 AM. Each ear was divided longitudinally into three equal sections: upper, middle, and lower. Kernels located at the boundary between the upper and the combined middle–lower sections were discarded. The upper kernels were pooled as inferior grains, and the middle–lower kernels were pooled as superior grains. After thorough mixing, the superior and inferior grains were separated, immediately frozen in liquid nitrogen, and stored at –80°C. The contents of indole–3–acetic acid (IAA), abscisic acid (ABA), and gibberellin (GA) in the grains were determined using specific ELISA kits: IAA (Kit EKPL408, Beijing Bio–Tech Pack Technology Company Ltd., Beijing), ABA (Kit EKPL241, Beijing Bio–Tech Pack Technology Company Ltd., Beijing), and GA (Kit EKPL104, Beijing Bio–Tech Pack Technology Company Ltd., Beijing), following the methods described by Wei et al. (2019) and Liang et al. (2017).

#### 2.2.3 Activities of enzymes related to grain starch synthesis

At anthesis, uniformly growing plants were selected and tagged. Every 12 days from 12 to 60 days after anthesis, three representative ears were collected from the tagged plants between 9:00 and 10:00 AM. Both superior and inferior grains were sampled from each ear, immediately frozen in liquid nitrogen and stored at –80°C. The activities of AGPase and SSS activities in the grains were measured according to the method of Yang et al. (2003).

#### 2.2.4 Grain starch accumulation and starch accumulation characteristics

Following plant tagging at anthesis, three representative ears were sampled every 12 days from 12 to 60 days after anthesis. For each sample, 100 superior and 100 inferior grains were oven–dried at 105°C for 30 min, then dried at 75°C to constant weight. The dried grains were weighed, ground, and their starch content was determined using the anthrone colorimetric method and were calculated for grain starch accumulation (Equation 1) and grain starch accumulation rate (Equation 2) (Yue et al., 2022b).

(1) Grain starch accumulation (g)=grain starch content (%)×grain dry weight (g)

(2) $$\text{Grain starch accumulation rate }(\text{g }100{\text{ grains}}^{–1}{\text{d}}^{–1})=[\text{Current grain starch accumulation }(\text{g})–\text{Previous grain starch accumulation }(\text{g})/\text{Number of days between measurements }(\text{d})$$

Grain accumulation parameters were simulated using a logistic equation, and the model was expressed as follows (Equation 3):

(3) $$y=a/(1+be^{–cx})$$

where a, b, and c were the fitted cumulative characteristic parameters. Based on these parameters, the following accumulation traits were calculated (Equations 4–8):

Amount of starch accumulated in the grain when the starch accumulation rate reaches its maximum.

(4) $$W_{\text{max}}(\text{g }100{\text{ grains}}^{–1})=0.5a$$

(5) $$\begin{array}{c} \text{Number of days required for the grain starch accumulation rate }\\ \text{to reach its maximum }{\text{T}}_{\text{max}}(\text{d})=\ln b/\text{c} \end{array}$$

(6) $$\text{Maximum grain starch accumulation rate }G_{\text{max}}(\text{g }100{\text{ grains}}^{–1}{\text{d}}^{–1})=[\text{c}\times W_{\text{max}}(\text{g }100{\text{ grains}}^{–1})]\times [1–(W_{\text{max}}(\text{g }100^{–1})/a)]$$

(7) $$\text{Average grain starch accumulation rate }G_{\text{mean}}(\text{g }100{\text{ grains}}^{–1}{\text{d}}^{–1})=ac/6$$

(8) $$\text{Active phase of grain starch accumulation }D(\text{d})=6/c_{{}^{\circ}}$$

#### 2.2.5 Changes in grain storage capacity and filling degree

Following plant tagging at anthesis, three representative ears were sampled every 12 days from 12 to 60 days after anthesis. For each sample, 100 superior and 100 inferior grains were oven–dried at 105°C for 30 min, then dried at 75°C to constant weight, after which the dry mass was measured. The grain–filling degree was then calculated as follows (Equation 9) (Zhang et al., 2020):

(9) Grain–filling degree=Dry weight per 100 grains/fresh volume per 100 grains

#### 2.2.6 Photosynthetic characteristics

From 0 to 48 days after anthesis, five representative plants were selected every 12 d from each plot. Net photosynthetic rate (P_n_), stomatal conductance (G_s_), intercellular CO_2_ concentration (C_i_), and transpiration rate (T_r_) in the middle of the ear–leaf were measured between 9:00 and 11:30 AM using a LI–6400 photosynthesis analyzer (LI–COR, USA).

#### 2.2.7 Chlorophyll content

Every 12 days from 0 to 48 days after anthesis, five representative plants were selected per plot. A 0.2 g segment from the mid–portion of the ear leaf was excised and extracted in 10 mL of 95% ethanol until complete discoloration. The absorbance of the supernatant was measured at 663 nm and 645 nm using a UV–1800 spectrophotometer. Chlorophyll content was calculated using the following formula (Equations 10–11) (Ren et al., 2016):

(10) $${\text{C}}_{\text{a}}=12.72\times {\text{OD}}_{663}–2.59{\text{OD}}_{645}$$

(11) $${\text{C}}_{\text{b}}=22.88\times {\text{OD}}_{645}–4.67\times {\text{OD}}_{663}$$

Where C_a_ and C_b_ represent the concentrations of chlorophyll a and chlorophyll b, respectively, in units of mg L^–1^.

#### 2.2.8 Leaf hormone content

Every 12 days from 0 to 48 days after anthesis, five representative plants were selected from each plot between 9:00 and 10:00 AM. The middle portion of the ear leaf was excised, immediately frozen in liquid nitrogen, and stored at –80°C. The contents of abscisic acid (ABA) and zeatin riboside (ZR) were determined using ELISA kits: ABA (Kit EKPL241, Beijing Bio–Tech Pack Technology Company Ltd., Beijing) and ZR (Kit EKPL5, Beijing Bio–Tech Pack Technology Company Ltd., Beijing), following the method described by Liu et al. (2011).

#### 2.2.9 Leaf antioxidant enzyme activity

Every 12 days from 0 to 48 days after anthesis, five representative plants were selected from each plot between 9:00 and 10:00 AM. The middle portion of the ear leaf was excised, immediately frozen in liquid nitrogen, and stored at –80°C. Superoxide dismutase (SOD) activity was determined by the nitro–blue tetrazolium photoreduction method, and peroxidase (POD) activity was measured according to Ren et al. (2023).

### 2.3 Statistical analysis

Data were organized using Microsoft Excel 2022. Prior to analysis, all datasets were tested for normality and homoscedasticity. Statistical analyses were performed with SPSS 26.0 (SPSS Inc., Chicago, IL, USA). One–way analysis of variance (ANOVA) was applied to assess differences among treatments, followed by Duncan’s multiple range test for *post hoc* comparisons. A p–value < 0.05 was considered statistically significant. Interaction effects were evaluated with nitrogen application rate (N) and year (Y) as main effects. Figures were prepared using Origin 2024. The grain starch accumulation process was modeled by curve fitting using Curve Expert 1.3, from which parameters A, B, and C were derived.

## 3 Results

### 3.1 Grain yield and yield components

Nitrogen fertilization (N) rates significantly affected kernel number per ear, 100–grain weight, and yield. Year (Y) significantly affected 100–grain weight and yield, and a significant interaction was observed between nitrogen fertilization rate (N) and year (Y). Notably, N180 treatment consistently achieved the highest grain yield in the growing seasons (2022–2024, **Table 1**). Compared with the N0, N90, N135, and N225 treatments, the N180 treatment increased yield by 55.7, 26.2, 16.4, and 5.8%, respectively. Nitrogen fertilization significantly increased the kernel number per ear and 100–grain weight, whereas no significant difference was detected in the number of ears per hectare. Specifically, kernel number per ear was 15.6, 10.6, and 6.1% higher in the N180 treatment than in the N0, N90, and N135 treatments, respectively. However, no significant difference was observed in kernel number per ear between the N180 and N225 treatments. Additionally, 100–grain weight was 37.9, 15.9, 11.2, and 4.4% higher in the N180 group than in the N0, N90, N135, and N225 treatment groups, respectively. Collectively, these results indicate that the N180 treatment attained the highest grain yield by increasing kernel number per ear and 100–grain weight.

**Table 1:** Grain yield and yield components.

Year	Treatment	Ear number (No./hm^2^)	Grain number per ear	100–grain weight (g)	Yield (kg ha^–1^)
2022	N0	59702a	549d	26.74e	8765.02e
	N90	59835a	573c	32.12d	11012.01d
	N135	59783a	597b	33.49c	11951.47c
	N180	59187a	639a	37.39a	14138.56a
	N225	59571a	628a	35.84b	13407.19b
2023	N0	59692a	555d	27.83e	9220.77e
	N90	59782a	583c	32.67d	11386.93d
	N135	59521a	607b	34.05c	12302.08c
	N180	58245a	644a	37.22a	13960.28a
	N225	57548a	633a	35.74b	13019.22b
2024	N0	61102a	558d	26.97e	9195.46e
	N90	59494a	581c	32.21d	11133.67d
	N135	59419a	606b	33.62c	12105.82c
	N180	58894a	638a	37.86a	14225.58a
	N225	59056a	637a	36.11b	13584.12b
ANOVA	N	ns	**	**	**
	Y	ns	ns	*	*
	N×Y	ns	ns	ns	**

N0 is the no–nitrogen fertilization rate during the entire reproductive period, and N90, N135, N180, and N225 represent nitrogen fertilization rates of 90, 135, 180, and 225 kg ha^–1^, respectively. Degrees of freedom for each test in the analysis of variance (ANOVA): between–group degrees of freedom = 2, within–group degrees of freedom = 30; values followed by different letters in the same column indicate significant differences at the 5% significance level. *indicates significance at the 0.05 probability level; **indicates significance at the 0.01 probability level.

### 3.2 Hormone content in superior and inferior grains

**Figure 1:**
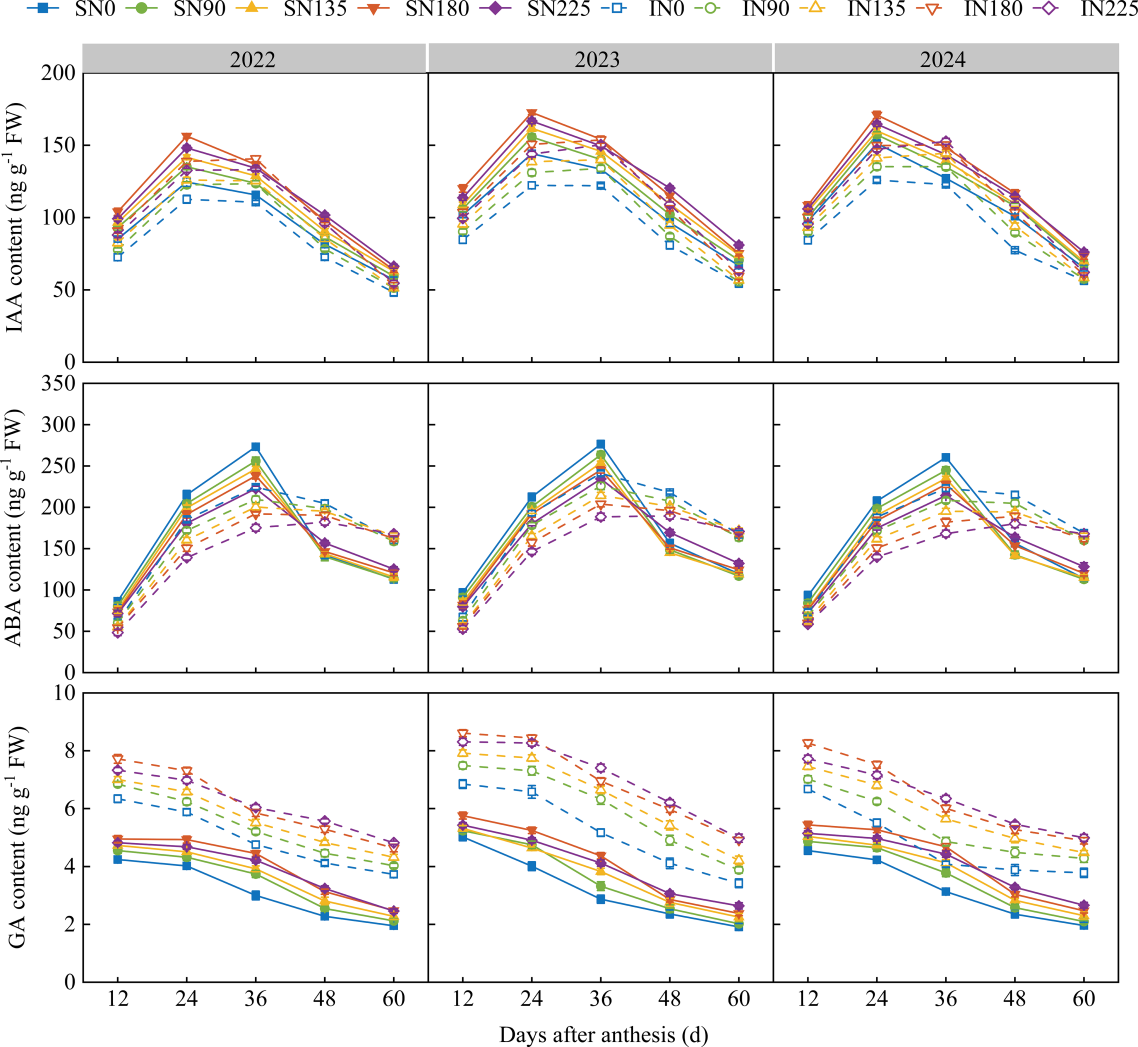
Effects of nitrogen fertilization rate on auxin (IAA), abscisic acid (ABA), and gibberellin (GA) contents in superior and inferior grains after anthesis. N0, N90, N135, N180, and N225 represent treatments with nitrogen fertilization rates of 0, 90, 135, 180 and 225 kg ha–1, respectively. 0, 12, 24, 36, 48, and 60 d represent 0, 12, 24, 36, 48, and 60 days after anthesis, respectively. 2022, 2023, and 2024 represent years 2022, 2023, and 2024, respectively.

From 2022 to 2024, both IAA and ABA content in superior and inferior grains exhibited a single–peak curve pattern of first increasing and then decreasing after anthesis (**Figure 1**). IAA and ABA contents in superior grains peaked at 24 and 36 days after anthesis, respectively, whereas the peaks occurred later in inferior grains and were lower than those in the superior grains. gibberellin (GA) content decreased gradually with increasing days after anthesis. At 12, 24, and 36 days after anthesis, the IAA content in both superior and inferior grains showed an initial increase, followed by a decrease with increasing nitrogen fertilization rates, with the N180 treatment exhibiting significantly higher levels than the other treatments. Compared with the N0, N90, N135, and N225 treatments, the N180 treatment increased average IAA content by 7.8, 11.2, 7.3, and 3.9%, respectively, in superior grains and by 23.9, 14.7, 8.9, and 3.6%, respectively, in inferior grains. At 48 and 60 days after anthesis, IAA content increased with increasing nitrogen fertilization rates, peaking in the N225 group. Additionally, ABA content at 24 and 36 days after anthesis decreased significantly with increasing nitrogen fertilization, with the highest and lowest values obtained in the N0 and N225 groups, respectively. At 48 days after anthesis, the ABA content in the superior grains was significantly higher in the N225 group than in the other groups. The GA content in inferior grains was more sensitive to nitrogen application rates than in superior grains. At 12 and 24 days after anthesis, it peaked in the N180 treatment, while at 36, 48, and 60 days after anthesis, the N225 treatment resulted in significantly higher GA content compared to the other treatments.

### 3.3 Enzyme activities in superior and inferior grains

**Figure 2:**
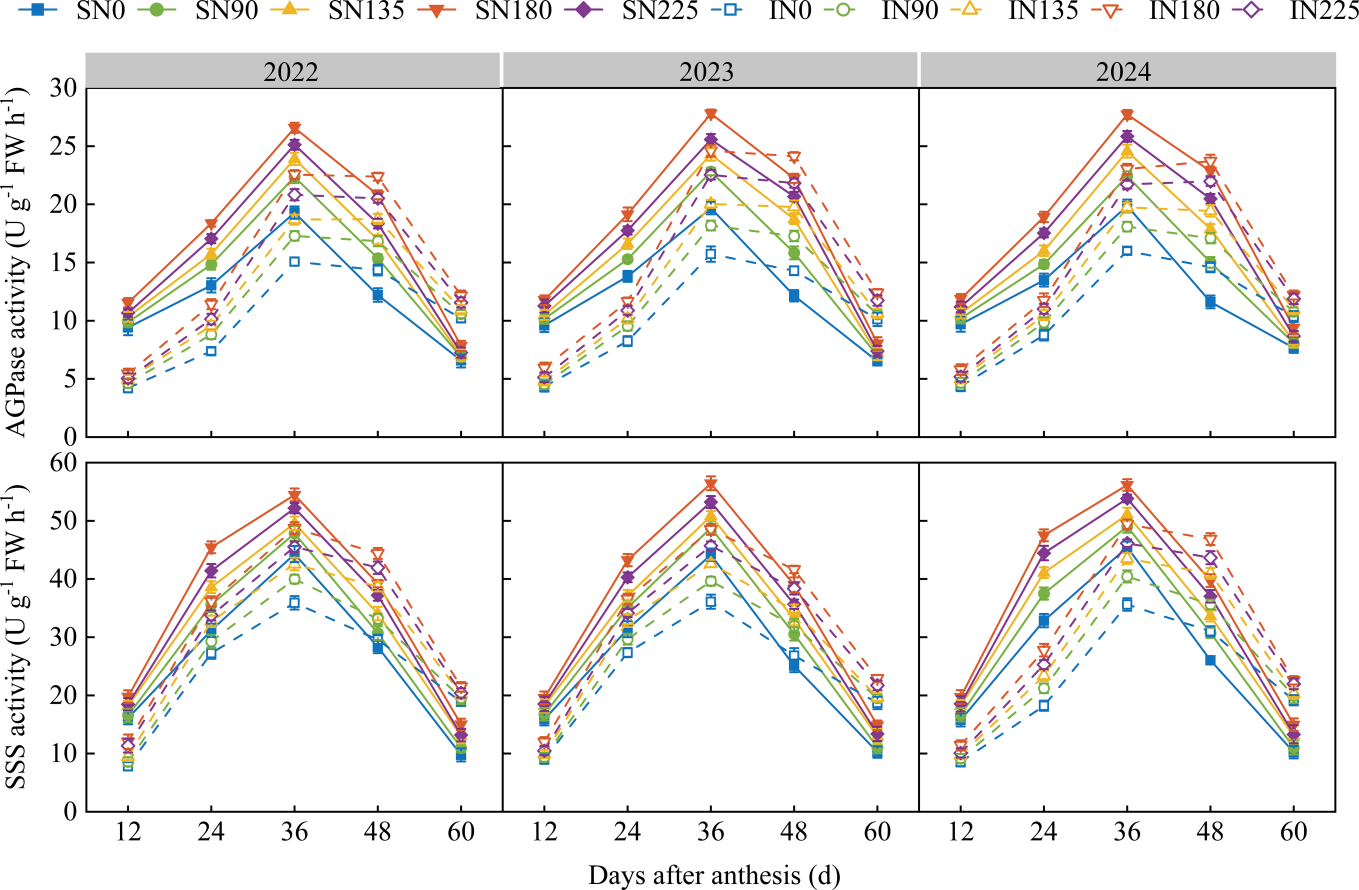
Effects of nitrogen fertilization rate on the activities of ADP–glucose pyrophosphorylase (AGPase) and soluble starch synthase (SSS) in superior and inferior grains after anthesis. N0, N90, N135, N180, and N225 represent treatments without nitrogen fertilization and with nitrogen fertilization rates of 0, 90, 135, and 180 kg ha^–1^, respectively. 0, 12, 24, 36, 48, and 60 d represent 0, 12, 24, 36, 48, and 60 days after anthesis, and 2022, 2023, and 2024 represent years 2022, 2023, and 2024, respectively.

During the study period (2022–2024), the activities of AGPase and SSS in both superior and inferior grains of maize after anthesis were significantly affected by nitrogen application rates. Both enzymes showed a unimodal curve pattern with increasing days after anthesis, peaking at 36 days after anthesis (**Figure 2**). With increasing nitrogen application rates, AGPase and SSS activities in both superior and inferior grains followed the order: N180 > N225 > N135 > N90 > N0. At the same sampling time after anthesis, the activities of AGPase and SSS were consistently higher in superior grains than in inferior grains. At 36 days after anthesis, AGPase activity in superior grains under the N180 treatment increased by 41.3%, 21.9%, 14.5%, and 8.8% compared to the N0, N90, N135, and N225 treatments, respectively, while SSS activity increased by 44.6%, 34.3%, 14.6%, and 11.0%, respectively. In inferior grains, the N180 treatment increased AGPase activity by 56.4%, 35.4%, 22.9%, and 9.2% and SSS activity by 22.4%, 17.4%, 16.6%, and 5.5% compared with the N0, N90, N135, and N225 treatments, respectively, at 36 days after anthesis. After 36 days after anthesis, AGPase and SSS activities declined rapidly in superior grains but decreased gradually in inferior grains.

### 3.4 Starch accumulation characteristics in superior and inferior grains

**Figure 3:**
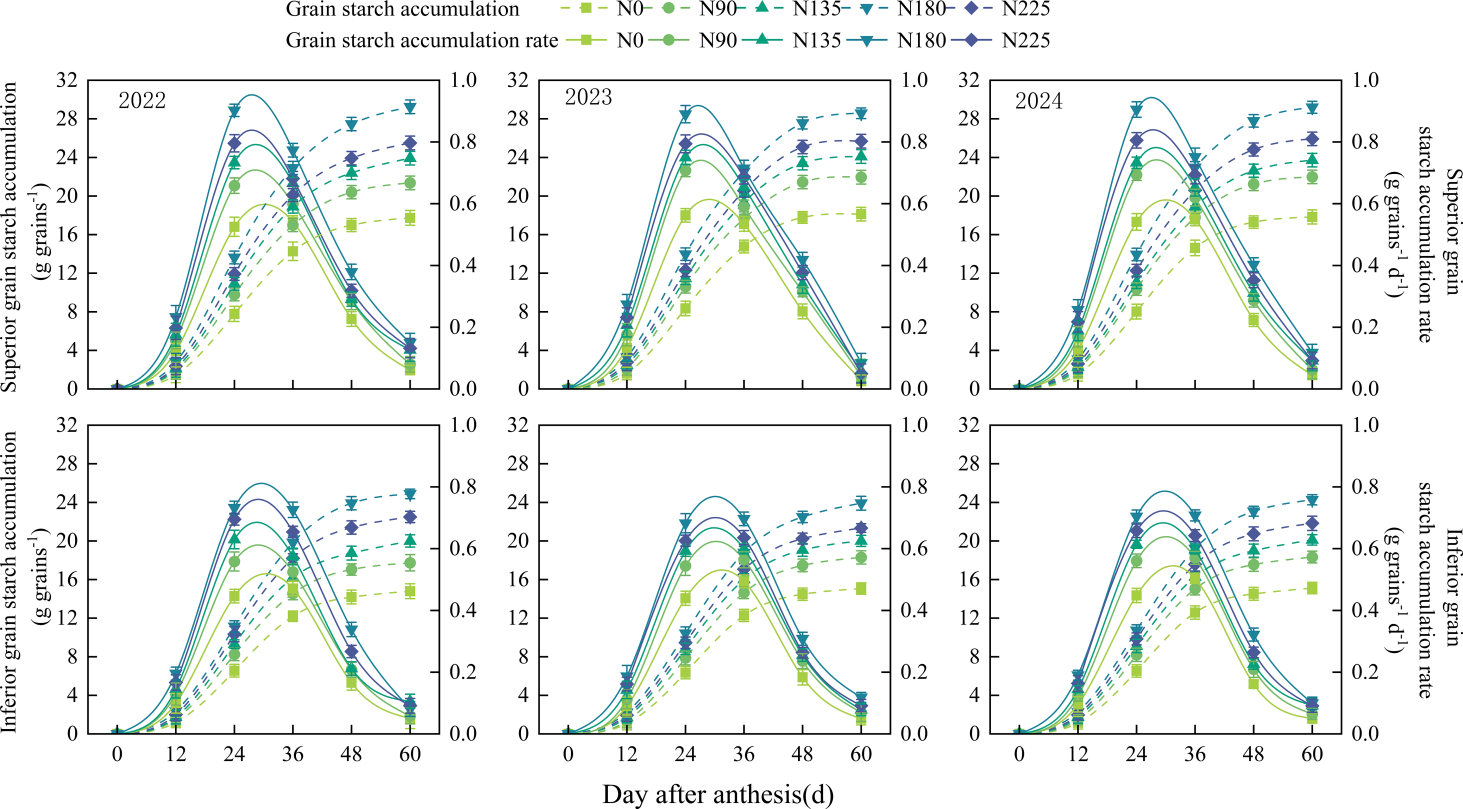
Effects of nitrogen fertilization rate on starch accumulation amount and accumulation rate in superior and inferior grains after anthesis. N0, N90, N135, N180, and N225 represent treatments with nitrogen fertilization rates of 0, 90, 135, 180 and 225 kg ha–1, respectively. 0, 12, 24, 36, 48, and 60 d represent 0, 12, 24, 36, 48, and 60 days after anthesis, and 2022, 2023, and 2024 represent years 2022, 2023, and 2024, respectively.

During the study period (2022–2024), no significant differences in starch accumulation were observed among the treatments at 12 days after anthesis (**Figure 3**). At 24 days after anthesis, starch accumulation in superior grains under the N180 treatment was significantly higher than that under the other treatments. While no significant differences were detected among the N90, N135, and N225 treatments, all of them resulted in significantly higher accumulation compared to the N0 treatment. In inferior grains, all nitrogen application treatments led to significantly greater starch accumulation than the N0 treatment. At 36, 48, and 60 days after anthesis, starch accumulation in both superior and inferior grains initially increased and then decreased with increasing nitrogen application rates, following the order: N180 > N225 > N135 > N90 > N0. At 60 days after anthesis, starch accumulation in superior grains under the N180 treatment was 27.7%, 30.2%, 18.6%, and 11.9% higher than that under the N0, N90, N135, and N225 treatments, respectively. Correspondingly, in inferior grains, it was 58.7%, 30.7%, 19.4%, and 12.0% higher than that under the N0, N90, N135, and N225 treatments, respectively. The starch accumulation rates in both superior and inferior grains across all treatments exhibited a unimodal curve pattern, with the N180 treatment showing the fastest rate, followed by the N225, N135, N90, and N0 treatments.

Nitrogen fertilization did not significantly alter the number of days required for the starch accumulation rate to reach its peak in either superior or inferior grains. However, the maximum accumulation rate of grain starch, the average starch accumulation rate, and the active accumulation period of grain starch all showed an initial increase followed by a decrease with increasing nitrogen application rates, with the N180 treatment yielding the highest values (**Table 2**). Additionally, all starch accumulation parameters were higher in superior grains than in inferior grains. Compared with the N0, N90, N135, and N225 treatments, the N180 treatment increased the maximum starch accumulation rate in superior grains by 57.5%, 30.3%, 18.6%, and 10.9%, respectively, and extended the active accumulation period of grain starch by 12.7%, 8.4%, 6.0%, and 4.1%, respectively. Over the three–year period, the average amount of starch accumulated when the accumulation rate peaked was 14.44 g. These findings indicate that the N180 treatment achieved highly efficient starch accumulation in grains by simultaneously optimizing both the accumulation rate and the duration of the active accumulation phase.

**Table 2:** Effects of nitrogen fertilization rate on starch accumulation parameters in superior and inferior grains.

Year	Item	Treatment	Growth curve parametric equation	Correlation coefficient	*T*_max_ (d)	*W*_max_ (g)	*G*_max_ (g d^–1^)	*G*_mean_ (g d^–1^)	D (d)
2022	superior grain	N0	*y* = 17.65/(1 + 53.38e^–0.15^*^x^*)	0.9991	26.00	8.82	0.67	0.45	39.22
		N90	*y* = 21.24/(1 + 44.15e^–0.15^*^x^*)	0.9984	25.71	10.62	0.78	0.52	40.73
		N135	*y* = 23.60/(1 + 42.73e^–0.15^*^x^*)	0.9980	25.71	11.80	0.86	0.57	41.08
		N180	*y* = 28.95/(1 + 36.91e^–0.14^*^x^*)	0.9977	25.63	14.47	1.02	0.68	42.61
		N225	*y* = 25.18/(1 + 39.01e^–0.14^*^x^*)	0.9977	25.51	12.59	0.90	0.60	41.77
	inferior grain	N0	*y* = 14.67/(1 + 63.22e^–0.16^*^x^*)	0.9994	25.65	7.34	0.59	0.40	37.12
		N90	*y* = 17.60/(1 + 52.65e^–0.16^*^x^*)	0.9989	25.26	8.80	0.69	0.46	38.24
		N135	*y* = 19.64/(1 + 48.91e^–0.15^*^x^*)	0.9982	25.23	9.82	0.76	0.50	38.91
		N180	*y* = 24.87/(1 + 44.16e^–0.15^*^x^*)	0.9991	25.93	12.43	0.91	0.61	41.07
		N225	*y* = 22.27/(1 + 48.39e^–0.15^*^x^*)	0.9987	25.45	11.14	0.85	0.57	39.37
2023	superior grain	N0	*y* = 18.18/(1 + 50.88e^–0.15^*^x^*)	0.9989	25.56	9.09	0.70	0.47	39.03
		N90	*y* = 21.97/(1 + 41.37e^–0.15^*^x^*)	0.9979	25.19	10.98	0.81	0.54	40.61
		N135	*y* = 24.13/(1 + 38.80e^–0.14^*^x^*)	0.9985	25.32	12.07	0.87	0.58	41.52
		N180	*y* = 28.63/(1 + 30.98e^–0.14^*^x^*)	0.9979	25.18	14.32	0.98	0.65	44.01
		N225	*y* = 25.81/(1 + 36.36e^–0.14^*^x^*)	0.9984	25.31	12.90	0.92	0.61	42.26
	inferior grain	N0	*y* = 14.99/(1 + 69.53e^–0.16^*^x^*)	0.9994	26.23	7.49	0.61	0.40	37.10
		N90	*y* = 18.18/(1 + 58.31e^–0.16^*^x^*)	0.9989	26.23	9.09	0.70	0.47	38.71
		N135	*y* = 19.89/(1 + 49.82e^–0.15^*^x^*)	0.9990	25.99	9.95	0.75	0.50	39.90
		N180	*y* = 23.76/(1 + 42.16e^–0.14^*^x^*)	0.9990	26.22	11.88	0.85	0.57	42.05
		N225	*y* = 21.19/(1 + 46.97e^–0.15^*^x^*)	0.9991	25.91	10.60	0.79	0.52	40.39
2024	superior grain	N0	*y* = 17.80/(1 + 56.06^–0.16^*^x^*)	0.9993	25.65	8.90	0.70	0.47	38.22
		N90	*y* = 21.90/(1 + 45.47e^–0.15^*^x^*)	0.9986	25.32	10.95	0.83	0.55	39.80
		N135	*y* = 23.58/(1 + 40.73e^–0.15^*^x^*)	0.9984	25.51	11.79	0.86	0.57	41.29
		N180	*y* = 29.08/(1 + 33.77e^–0.14^*^x^*)	0.9979	25.40	14.54	1.01	0.67	43.31
		N225	*y* = 25.82/(1 + 37.61e^–0.14^*^x^*)	0.9983	25.41	12.91	0.92	0.61	42.03
	inferior grain	N0	y = 15.00/(1 + 73.70e^–0.17^*^x^*)	0.9996	25.79	7.50	0.63	0.42	35.99
		N90	y = 18.17/(1 + 60.13e^–0.16^*^x^*)	0.9992	25.61	9.08	0.73	0.48	37.51
		N135	y = 19.86/(1 + 50.68e^–0.15^*^x^*)	0.9988	25.54	9.93	0.76	0.51	39.04
		N180	y = 24.19/(1 + 43.21e^–0.14^*^x^*)	0.9991	26.07	12.09	0.87	0.58	41.54
		N225	y = 21.65/(1 + 47.61e^–0.15^*^x^*)	0.9989	25.67	10.82	0.81	0.54	39.88

N0, N90, N135, N180, and N225 represent treatments with nitrogen fertilization rates of 0, 90, 135, 180 and 225 kg ha–1, respectively. where A, B, and C are model parameters; T_max_ is the number of days required for the grain starch accumulation rate to peak; W_max_ represents the grain starch accumulation amount when the grain starch accumulation rate peaked; G_max_ denotes the maximum accumulation rate of grain starch; G_mean_ indicates the average starch accumulation rate in the grain; and D is the active accumulation period of grain starch.

### 3.5 Changes in grain sink capacity and filling

**Figure 4:**
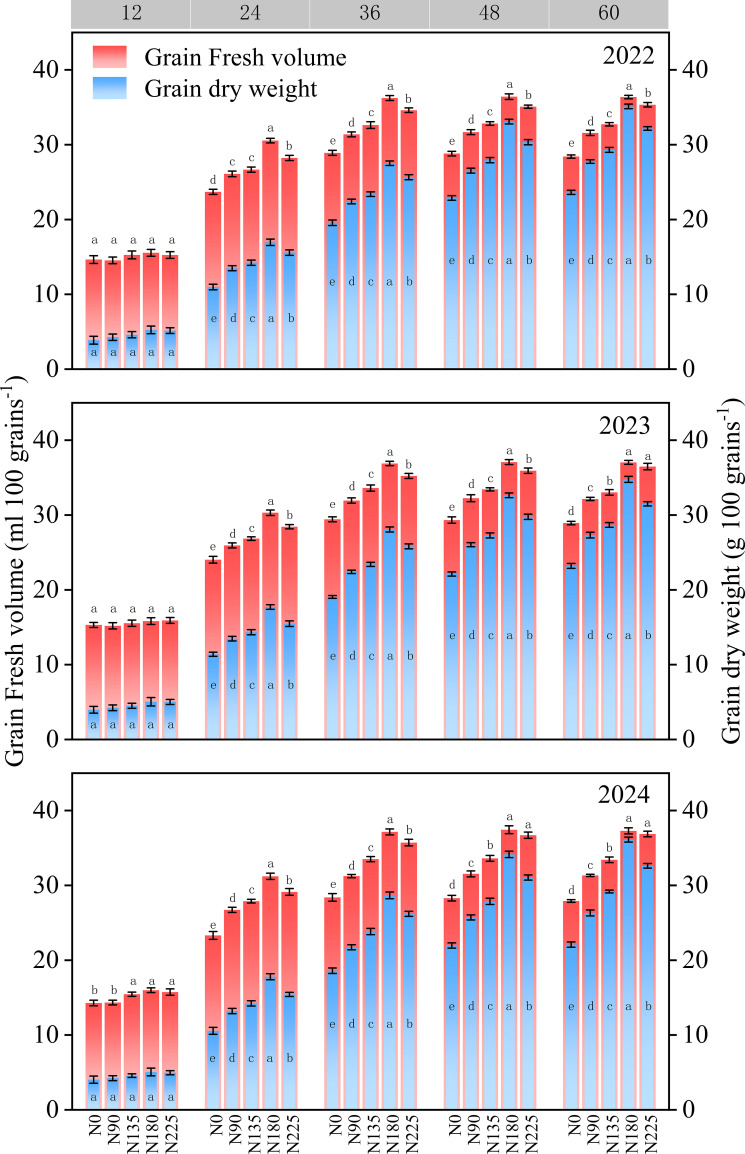
Effects of nitrogen fertilization rate on grain volume and grain weight after anthesis. N0, N90, N135, N180, and N225 represent treatments with nitrogen fertilization rates of 0, 90, 135, 180 and 225 kg ha–1, respectively. 0, 12, 24, 36, 48, and 60 d represent 0, 12, 24, 36, 48, and 60 days after anthesis, and 2022, 2023, and 2024 represent years 2022, 2023, and 2024, respectively.

Both fresh grain volume and grain dry weight increased continuously throughout grain development, with the fresh grain volume stabilizing at 36 days after anthesis (**Figure 4**). At 12 days after anthesis, no significant differences were observed in grain fresh volume or grain dry weight among the treatments, except in 2024, when the N0 and N90 treatments exhibited significantly lower grain fresh volume than the N135, N180, and N225 treatments. At 24, 36, 48, and 60 days after anthesis, both fresh grain volume and dry weight initially increased and then decreased with increasing nitrogen fertilization rates, with the highest values observed in the N180 group. The average fresh volume of grains was 26.5%, 15.6%, 11.3%, and 3.9% higher than that of the N0, N90, N135, and N225 treatments, respectively. The grain dry weight was 49.4%, 26.8%, 20.8%, and 10.4% higher, respectively.

**Figure 5:**
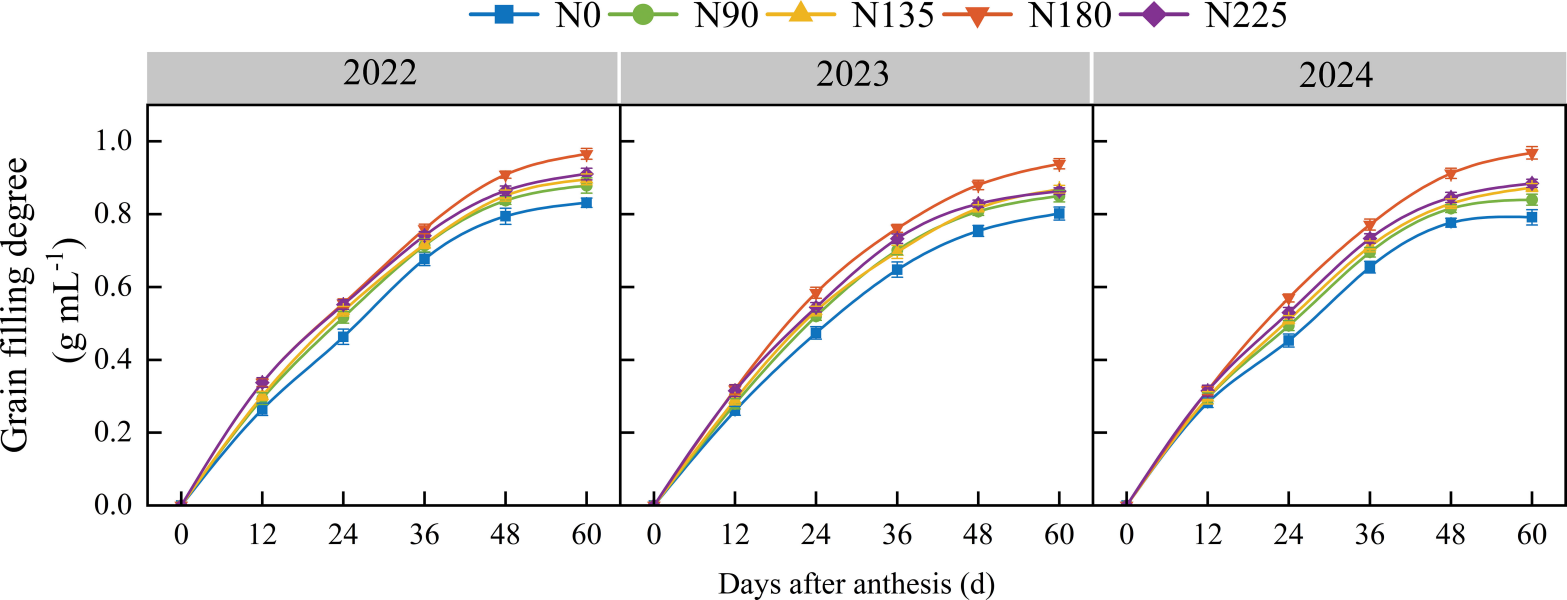
Effects of nitrogen fertilization rate on grain filling degree. N0, N90, N135, N180, and N225 represent treatments with nitrogen fertilization rates of 0, 90, 135, 180 and 225 kg ha–1, respectively. 0, 12, 24, 36, 48, and 60 d represent 0, 12, 24, 36, 48, and 60 days after anthesis, and 2022, 2023, and 2024 represent years 2022, 2023, and 2024, respectively.

From 2022 to 2024, the degree of grain filling under different nitrogen fertilization treatments initially increased rapidly and then stabilized with increasing days after anthesis (**Figure 5**). The N180 treatment consistently showed the highest grain–filling degree, which was 18.6%, 10.3%, 8.7%, and 6.0% higher than that in the N0, N90, N135, and N225 treatments, respectively. At 36 days after anthesis, the grain–filling degree in the N225 treatment was significantly lower than that in the N180 treatment by 6.4%, while the N0 treatment maintained the lowest grain–filling degree throughout the period.

### 3.6 Photosynthetic characteristics of ear leaf

**Figure 6:**
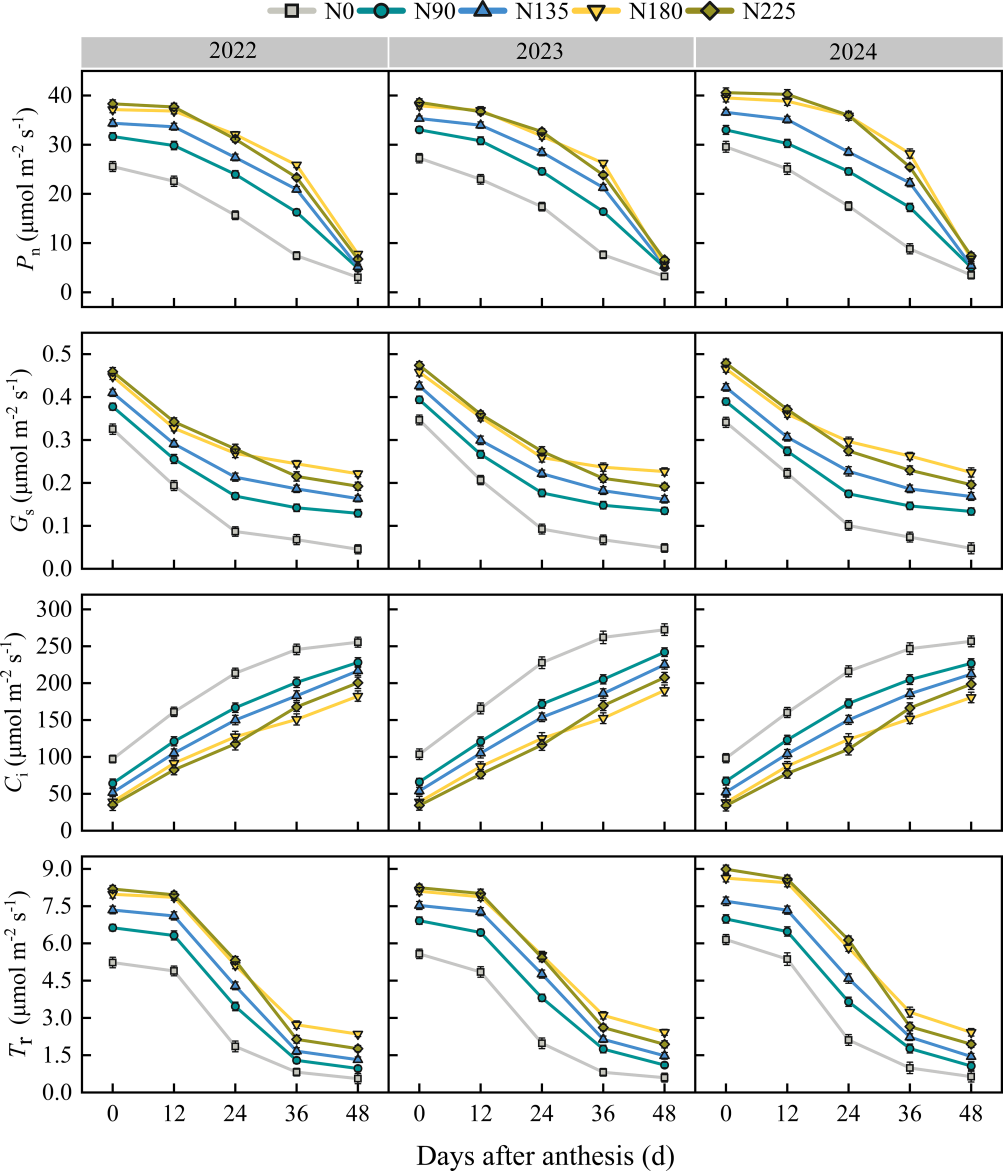
Effects of nitrogen fertilization rate on the photosynthetic characteristics of the ear leaf after anthesis. N0, N90, N135, N180, and N225 represent treatments with nitrogen fertilization rates of 0, 90, 135, 180 and 225 kg ha–1, respectively. 0, 12, 24, 36, 48, and 60 d represent 0, 12, 24, 36, 48, and 60 days after anthesis, and 2022, 2023, and 2024 represent years 2022, 2023, and 2024, respectively.

During the study period (2022–2024), the P_n_, G_s_, and T_r_ of ear leaves of post–anthesis maize gradually decreased with the progress of growth, while C_i_ gradually increased (**Figure 6**). At 0, 12, and 24 days after anthesis, the N180 treatment exhibited significantly higher P_n_, G_s_, and T_r_ values than the N0, N90, and N135 treatments, with no significant differences observed between the N180 and N225 treatments. C_i_ was highest in the N0 treatment, followed by the N90, N135, N180, and N225 treatments, with no significant difference between the N180 and N225 treatments. At 36 days after anthesis, the P_n_ of N180 treatment was significantly better than other treatments, while there was no significant difference in P_n_ among the nitrogen application treatments at 48 days after anthesis, all of which were higher than the N0 treatment. Furthermore, at 36 and 48 days after anthesis, G_s_ and T_r_ were highest in the N180 treatment, followed by the N225 treatment. In contrast, C_i_ showed an opposite trend: N0 > N90 > N135 > N225 > N180.

### 3.7 Chlorophyll content in the ear leaf

**Figure 7:**
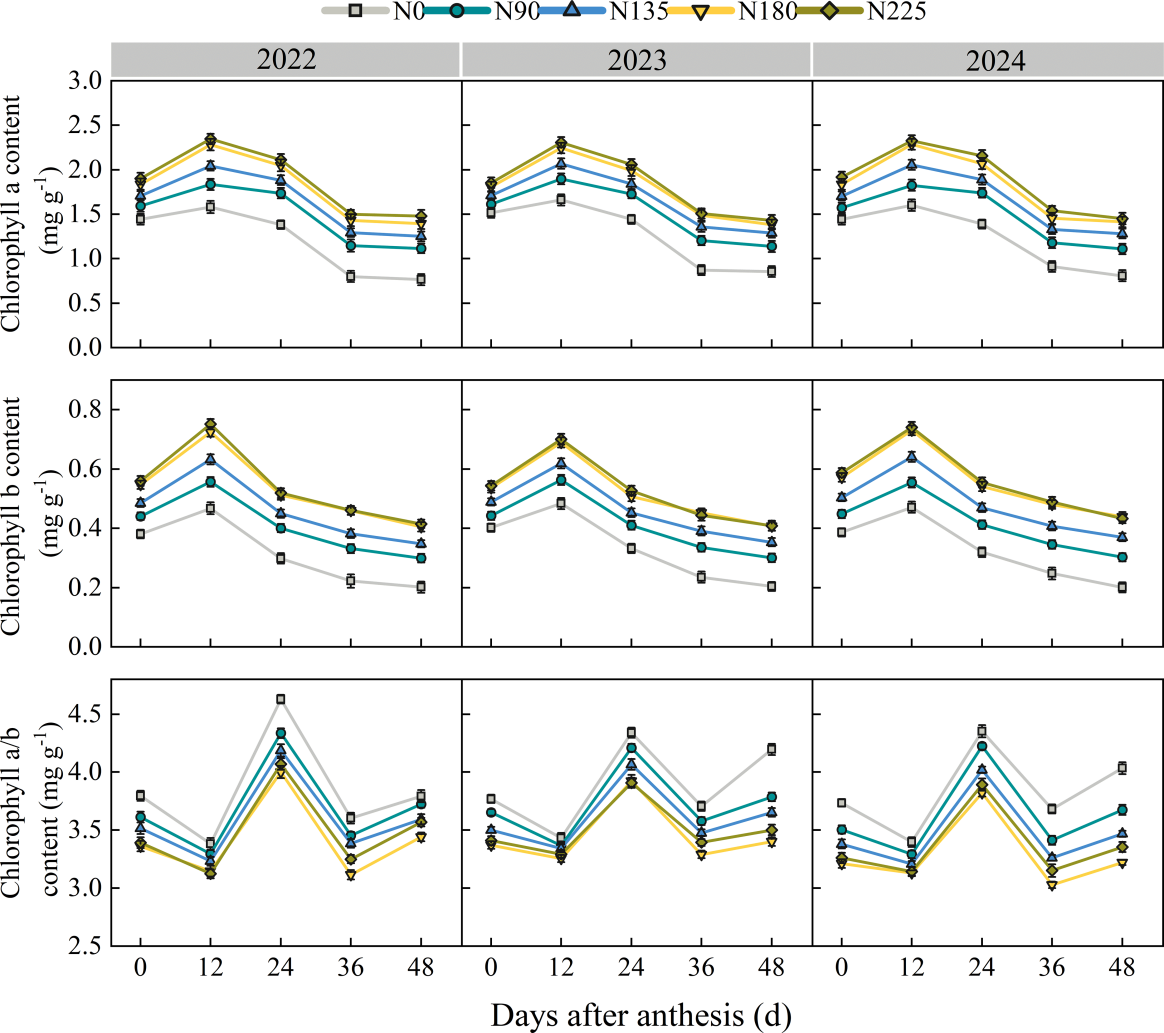
Effects of nitrogen fertilization rate on chlorophyll content in the ear leaf after anthesis. N0, N90, N135, N180, and N225 represent treatments with nitrogen fertilization rates of 0, 90, 135, 180 and 225 kg ha–1, respectively. 0, 12, 24, 36, 48, and 60 d represent 0, 12, 24, 36, 48, and 60 days after anthesis, and 2022, 2023, and 2024 represent years 2022, 2023, and 2024, respectively.

From 2022 to 2024, as the number of days after anthesis increases, the content of chlorophyll a (Chl a) and chlorophyll b (Chl b) in each treatment shows a trend of first increasing and then decreasing, reaching its peak at 12 days after anthesis (**Figure 7**). At 0, 12, 24, 36, and 48 days after anthesis, the Chl a and Chl b contents in the N180 treatment were significantly higher than those in the N0, N90, and N135 treatments, with no significant difference compared to the N225 treatment. At 0, 12, and 24 days after anthesis, the average chlorophyll a/b (Chl a/b) of N180 treatment was 8.6%, 6.0%, and 3.3% lower than that of N0, N90, and N135 treatments, respectively, with no significant difference compared to N225 treatment. At 36 and 48 days after anthesis, the Chl a/b ratio of N180 treatment was significantly lower than that of other treatments.

### 3.8 Hormone contents in ear leaf

**Figure 8:**
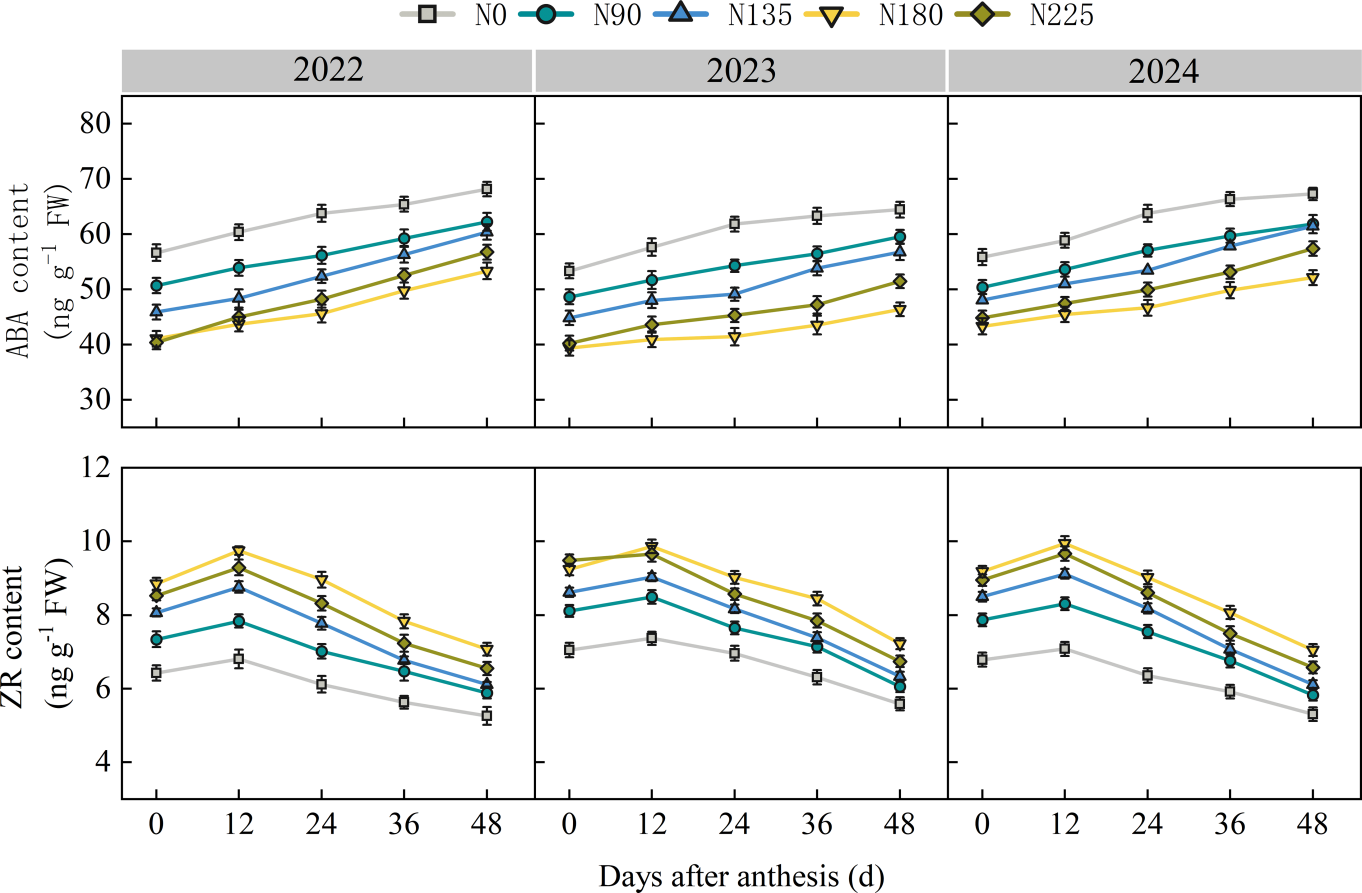
Effects of nitrogen fertilization rate on the contents of abscisic acid (ABA) and zeatin riboside (ZR) in ear leaf after anthesis. N0, N90, N135, N180, and N225 represent treatments with nitrogen fertilization rates of 0, 90, 135, 180 and 225 kg ha–1, respectively. 0, 12, 24, 36, 48, and 60 d represent 0, 12, 24, 36, 48, and 60 days after anthesis, and 2022, 2023, and 2024 represent years 2022, 2023, and 2024, respectively.

From 2022 to 2024, the ABA content in the ear leaf gradually increases with the advancement of the growth process after anthesis, while the ZR content shows a unimodal curve and reaches its peak 12 days after anthesis (**Figure 8**). At 0 and 12 days after anthesis, the ABA content was higher in the N0, N90, and N135 treatments, while there was no significant difference between the N180 and N225 treatments and they both maintained the lowest level; The ZR content was significantly higher in N180 and N225 treatments than in N135, N90, and N0 treatments. At 24, 36, and 48 days after anthesis, the ABA content was highest in the N0 treatment and lowest in the N180 treatment. The N225 treatment increased by 9.5% compared to the N180 treatment; The ZR content was highest in the N180 treatment, with an increase of 6.6% compared to the N225 treatment. This indicates that N180 treatment can maintain hormone balance in ear leaf after anthesis and delayed leaf senescence.

### 3.9 Enzyme activities in the ear leaf

**Figure 9:**
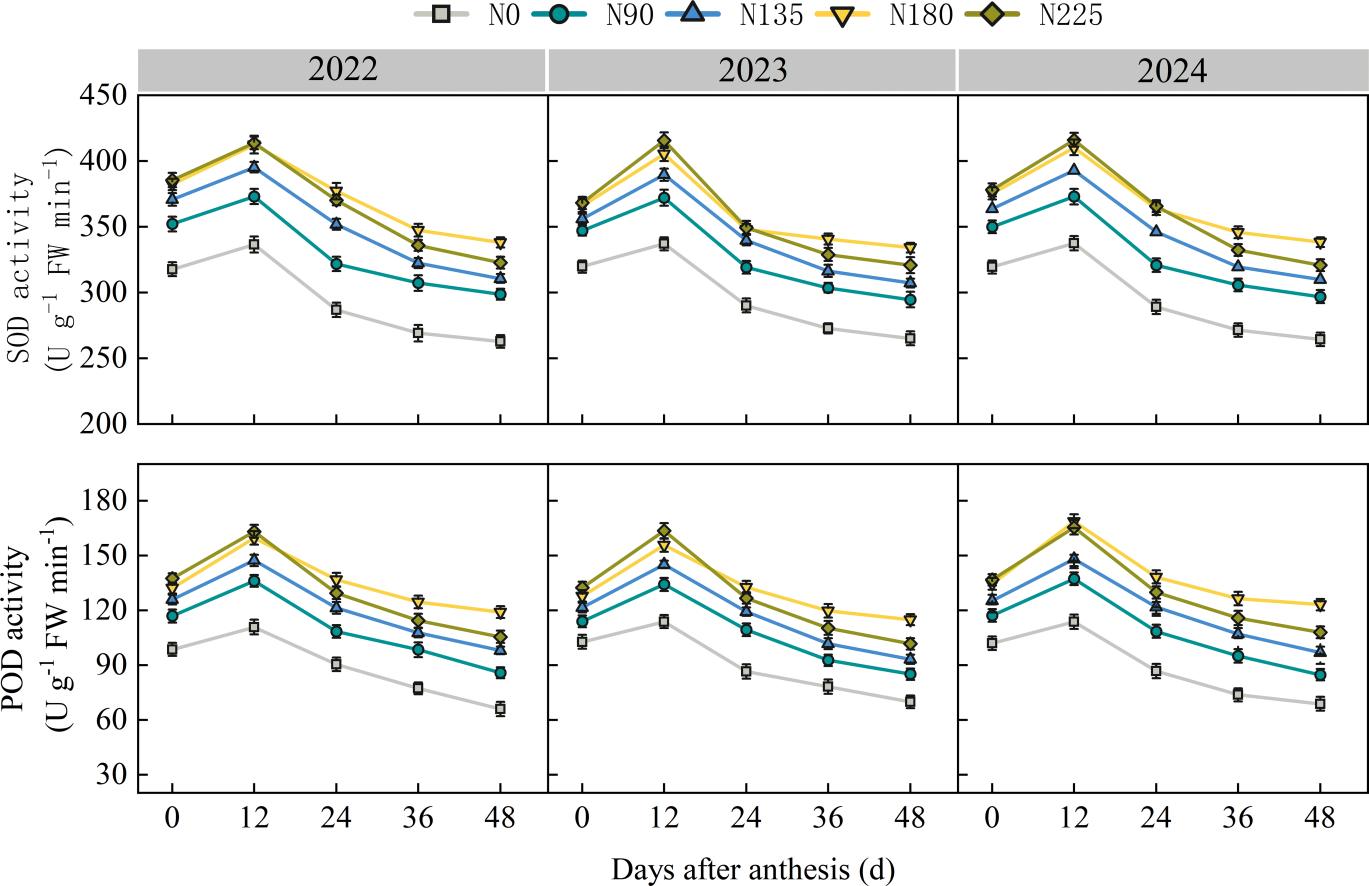
Effects of nitrogen fertilization rate on the activities of superoxide dismutase (SOD) and peroxidase (POD) in ear leaf after anthesis. N0, N90, N135, N180, and N225 represent treatments with nitrogen fertilization rates of 0, 90, 135, 180 and 225 kg ha–1, respectively. 0, 12, 24, 36, 48, and 60 d represent 0, 12, 24, 36, 48, and 60 days after anthesis, and 2022, 2023, and 2024 represent years 2022, 2023, and 2024, respectively.

From 2022 to 2024, SOD and POD activities in maize ear leaves exhibited unimodal curves during the post–anthesis growth process, peaking at 12 days after anthesis (**Figure 9**). At 0, 12, and 24 days after anthesis, SOD and POD activities were highest in the N180 and N225 treatments, with no significant difference between the two treatments. Compared with N0, N90, and N135 treatments, N180 treatment increased SOD and POD activities by 18.3%, 7.8%, and 3.1%, and 37.4%, 16.5%, and 7.9%, respectively. At 36 and 48 days after anthesis, SOD and POD activities across treatments increased with nitrogen application rate, following the order N180 > N225 > N135 > N90 > N0. SOD activity in the N225 treatment decreased by 3.9% and POD activity decreased by 10.7% compared to N180, indicating that the N225 treatment accelerated the decline in antioxidant enzyme activity, leading to latent senescence in the ear leaves.

## 4 Discussion

### 4.1 Effects of nitrogen fertilization rate on maize yield and its components

Achieving higher maize yield relies on coordinated improvements in yield components (Wu et al., 2024). Previous studies have shown that nitrogen fertilization primarily regulates grain yield by influencing kernel number per ear and 100–kernel weight (Hou et al., 2019). In this study, the yield of maize showed a trend of first increasing and then decreasing with increasing nitrogen application rate. When the nitrogen application rate was 180 kg ha^–1^, the grain yield reached its maximum, with a three–year average increase of 55.7%, which was due to the coordinated improvement of kernel number per ear and 100–kernel weight. Previous research has indicated that kernel number per ear increases with increasing nitrogen rates within a nitrogen application range of 90–270 kg ha^–1^; however, it plateaus or even declines at higher nitrogen application rates (Jiang et al., 2019). Similarly, our study showed that an increase in nitrogen application to 225 kg ha^–1^ did not significantly increase kernel number per ear but markedly decreased 100–kernel weight, ultimately leading to a reduced yield. This may be because moderate nitrogen levels promote tassel and ear differentiation and increase kernel number by regulating proteins and genes related to fatty acid metabolism, thereby enhancing fatty acid synthesis and elongation. However, increasing the nitrogen application rate on this basis does not continuously increase the synthesis rate of fatty acids, and therefore cannot continue to increase the kernel number per ear (Yu et al., 2023a). Grain weight formation is highly dependent on the continuous supply of photoassimilates after anthesis and efficient grain sink filling (Wang et al., 2024b). Our results further demonstrate that yield improvement in maize depends not only on the direct regulation of yield components by nitrogen fertilization but also on the coordinated optimization of “source” and “sink” (Yan et al., 2022). In terms of the “source,” the N180 treatment delayed leaf senescence by maintaining higher chlorophyll content, photosynthetic rate, and antioxidant enzyme activities, thereby ensuring a continuous supply of photoassimilates for grain development. From the perspective of the “sink,” this treatment significantly enhanced the activities of key starch–synthesizing enzymes, optimized the hormonal balance between superior and inferior grains, and improved starch accumulation capacity in both grain types, thereby significantly increasing 100–kernel weight and establishing a foundation for high yield. In contrast, although the N225 treatment increased kernel number per ear to some extent, it induced premature leaf senescence during the late filling stage and weakened sink activity, leading to a reduction in kernel weight and thereby limiting yield improvement. In addition, studies conducted under different soil fertility levels have shown that maize yield and nitrogen fertilizer recovery efficiency exhibit a significant quadratic response to nitrogen fertilization rate. The theoretical yield reaches its maximum when the nitrogen fertilization rate ranges from 126.9 to 181.8 kg ha^–1^, whereas the actual nitrogen application rate required to achieve 95% of the maximum yield and optimal nitrogen recovery efficiency is usually lower than this range (Zou et al., 2024). Notably, high maize yield and nitrogen fertilizer use efficiency do not rely on high nitrogen application rates. Long–term field trials have demonstrated that the yield of spring maize first increases and then stabilizes with increasing nitrogen application rates. However, when the nitrogen application rate exceeds 225 kg ha^–1^, the residual nitrate nitrogen in the soil increases significantly, and nitrogen fertilizer use efficiency decreases markedly (Ji et al., 2025). In this study, the optimal nitrogen fertilization rate (N180) proved to be an effective fertilization strategy for reducing nitrogen use, enhancing efficiency, and promoting the sustainability of maize cultivation. This was achieved by optimizing the source–sink relationship and coordinating the increases in both grain number per ear and hundred–grain weight, thereby boosting yield.

### 4.2 Effects of nitrogen fertilization rate on leaf photosynthetic performance

Improving leaf photosynthetic performance is key to achieving high maize yields, with the light capture efficiency of leaves largely dependent on the content of photosynthetic pigments such as chlorophyll (Sun et al., 2023). Therefore, the content and dynamic changes in chlorophyll a (Chl a) and chlorophyll b (Chl b) are crucial for light–energy conversion (Li et al., 2024a). In the present study, N180 and N225 treatments significantly increased Chl a and b contents after anthesis compared with N0, N90, and N135 treatments. This stems from the critical role of nitrogen in maintaining chloroplast structure, which ensures normal chloroplast division and orderly stacking of grana lamellae, and provides a structural foundation for chlorophyll synthesis (Cheng et al., 2025; Li et al., 2013). Moreover, nitrogen application reduces chlorophyll a/b (Chl a/b) ratio, which enhances the ability of leaves to absorb short–wavelength light and promotes photosynthetic carbon assimilation and dry matter accumulation in grains (Li et al., 2023). In the present study, during the early and middle grain–filling stages, the Chl a/b ratios under the N180 and N225 treatments were significantly lower than those under the other treatments, with no significant difference between the two treatments, which is consistent with the findings mentioned above. However, during the late grain–filling stage, the Chl a/b ratio under the N225 treatment was significantly higher than that under the N180 treatment. Although no significant difference in chlorophyll content was observed between the two treatments, the net photosynthetic rate (P_n_) of the N225 treatment decreased by 16.66% compared with that of the N180 treatment, accompanied by reduced stomatal conductance (G_s_) and increased intercellular CO_2_ concentration (C_i_). This indicates that the N225 treatment induced covert senescence in plants during the late post–anthesis stage, meaning that although the leaves remained green morphologically, their photosynthetic function was already impaired. Recent field studies have also found that maize has an optimal nitrogen application range. Appropriate nitrogen rates can significantly increase yield by delaying ear leaf senescence and improving nitrogen use efficiency, whereas either excessive or insufficient nitrogen application shortens the functional duration of the ear leaf (Du et al., 2024), which aligns with the results of this study.

The leaf senescence process after maize anthesis is closely associated with the accumulation of reactive oxygen species (ROS). The activities of key antioxidant enzymes, superoxide dismutase (SOD) and peroxidase (POD), reflect the degree of leaf senescence (Hu et al., 2023). In this study, both the N180 and N225 treatments maintained relatively high SOD and POD activities during the early and middle grain–filling stages, which may be related to the role of nitrogen in promoting the synthesis of enzyme proteins (Zhang et al., 2024). However, during the late grain–filling stage, enzyme activities were significantly lower in the N225 treatment than in the N180 treatment, indicating that excessive nitrogen application weakens the leaf antioxidant system. This can lead to excessive ROS accumulation, thereby accelerating leaf senescence. Previous studies have pointed out that when the balance between ROS production and scavenging mechanisms is disrupted, excess ROS acts both as a signal and as a damaging factor to promote leaf senescence (Rao et al., 2025). Premature senescence in rice under nitrogen–stress conditions is accompanied by decreased antioxidant enzyme activity, excessive ROS accumulation, and increased ABA content (Zakari et al., 2020). Furthermore, studies have shown that elevated ABA is not only a signal of nutrient stress but can also accelerate leaf senescence by regulating ROS metabolism and inducing the expression of senescence–related genes (Li et al., 2022). In terms of hormonal regulation, cytokinin–type hormones such as zeatin riboside (ZR) can delay leaf senescence by suppressing the expression of senescence–related genes and maintaining chloroplast structure, whereas abscisic acid (ABA) promotes chlorophyll degradation and senescence by activating senescence signaling pathways (Li et al., 2024b). In this study, both the N180 and N225 treatments exhibited high ZR and low ABA levels during the early grain–filling stage, consistent with the role of nitrogen in promoting cytokinin synthesis and inhibiting ABA metabolism (Zhao et al., 2023). However, during the mid– to late–filling stages, only the N180 treatment maintained relatively high ZR and low ABA levels, while the N225 treatment showed a decline in ZR and a relative accumulation of ABA, indicating that excessive nitrogen application disrupted the ZR/ABA balance. Therefore, excessive nitrogen application (N225) likely triggered premature–senescence signals by weakening the antioxidant system, inducing ABA accumulation, and disrupting the ZR/ABA balance during the mid–to late–filling stages, leading to an earlier decline in leaf photosynthetic function (Lv et al., 2021). In contrast, the appropriate nitrogen rate (N180) delayed post–anthesis leaf functional senescence by maintaining stronger antioxidant enzyme activity alongside a balanced ZR/ABA ratio. Future studies could further verify this mechanism using indicators such as membrane lipid peroxidation, chloroplast ultrastructure, and the expression of senescence–related genes.

### 4.3 Effects of nitrogen fertilization rate on starch accumulation dynamics in superior and inferior grains

Starch is the primary component of maize grains and the grain–filling process is essentially a process of starch synthesis and accumulation (Hu et al., 2021). Therefore, the dynamics of starch accumulation in grains are crucial for maize yield. Studies have shown that appropriately increasing nitrogen application (N240) can enhance grain starch content (Liu et al., 2025). By fitting the starch accumulation characteristics of superior and inferior grains under different nitrogen application rates using a logistic equation, this study found that the optimal nitrogen rate (N180) achieved the highest starch accumulation in both grain types by maintaining a high starch accumulation rate while extending the active accumulation period. This result is consistent with previous findings that nitrogen application affects kernel weight by regulating the grain–filling rate and the effective filling duration. The activity levels of key starch–synthesizing enzymes are important indicators reflecting grain sink activity (Liu et al., 2021). The activities of ADP–glucose pyrophosphorylase (AGPase) and soluble starch synthase (SSS) are positively correlated with the starch synthesis rate (V et al., 2019). Research has indicated that higher nitrogen application levels (200 and 300 kg ha^–1^) can significantly increase AGPase and SSS activities, leading to a 10.44–10.86% increase in grain starch accumulation compared to the no–nitrogen treatment (Yue et al., 2022a). This demonstrates that an appropriate nitrogen rate promotes starch accumulation by enhancing the activities of key starch–synthesizing enzymes. Another study noted that AGPase activity initially increases and then decreases with increasing nitrogen application, peaking at 240 kg ha^–1^ (Feng et al., 2024). In the present study, the AGPase and SSS activities in both superior and inferior grains were highest under the appropriate nitrogen application rate (N180), and the peak time of enzyme activity was highly consistent with the peak rate of starch accumulation. This indicates that this nitrogen level maintained a high enzymatic synthesis capacity during the critical filling stage, providing a direct driving force for rapid starch accumulation in grains and serving as an important physiological basis for increasing starch accumulation in both superior and inferior grains. This could be related to the fact that an adequate nitrogen supply promotes plant nitrogen metabolism and amino acid synthesis, which provides sufficient substrates for the synthesis of enzymatic proteins, thereby enhancing enzyme activity (Yu et al., 2023b). Studies have shown that under different nitrogen levels, the activities of sucrose synthase and AGPase, as well as the average starch accumulation rate, are significantly higher in superior grains than in inferior grains (Li et al., 2021). This study further revealed that the peak AGPase and SSS activities in superior grains occurred earlier and were higher, providing a direct physiological basis for their earlier filling initiation and higher starch accumulation rate. In contrast, the peak enzyme activity in inferior grains was delayed and lower, resulting in weaker starch synthesis capacity during the early and middle filling stages. Moreover, the response amplitude of starch accumulation parameters in inferior grains to nitrogen application was greater than that in superior grains, suggesting that optimizing nitrogen application to improve the filling of inferior grains is a feasible strategy for further enhancing maize yield.

Endogenous hormones play a central role in regulating grain sink capacity and sink strength in maize, and their dynamic changes directly influence grain development and starch accumulation (Teng et al., 2022). Auxin (IAA) primarily enhances grain sink capacity and strength by promoting endosperm cell division and proliferation. Gibberellin (GA), in synergy with IAA, favors cell elongation and dry matter accumulation (Liu et al., 2024; Yu et al., 2024); however, excessively high GA levels can enhance the activity of starch hydrolases, accelerate starch degradation, and thereby inhibit starch accumulation (Yu et al., 2024). An appropriate amount of abscisic acid (ABA) helps promote the synthesis of storage substances and grain filling, but excessively high ABA content may antagonize other hormones such as IAA and inhibit sucrose transport to grains, hindering starch synthesis (Yu et al., 2020). Research has indicated that as the nitrogen application rate increased from 0 to 184.5 kg ha^–1^, the contents of IAA and zeatin riboside (ZR) in grains gradually rose, whereas further increasing the nitrogen rate to 300 kg ha^–1^ led to a significant reduction in both. During the mid to late grain–filling stages, the ABA content was lowest at the nitrogen rate of 184.5 kg ha^–1^, which was significantly lower than that under the 0, 129, and 300 kg ha^–1^ treatments. The results demonstrate that the appropriate nitrogen rate (184.5 kg ha^–1^) achieved an optimal endogenous hormone balance by synergistically elevating IAA and ZR levels while suppressing excessive ABA accumulation, thereby increasing the maximum grain–filling rate and extending the active filling period, which significantly enhanced kernel weight (Yu et al., 2020). In this study, the appropriate nitrogen rate (N180) significantly increased IAA and GA contents and reduced ABA content during the early filling stage, which was beneficial for promoting endosperm cell division and expansion, enlarging grain sink capacity, enhancing the competitiveness of grains for assimilates and their starch synthesis capacity, thus laying the foundation for subsequent rapid starch accumulation. Insufficient or excessive nitrogen application disrupted the hormonal balance. On one hand, it led to excessively high GA and ABA contents during the mid–to–late filling stages, potentially enhancing starch–degrading enzyme activity and accelerating starch hydrolysis. On the other hand, excessively high ABA content inhibited the transport of assimilates to grains, ultimately hindering normal starch accumulation and grain filling, resulting in decreased sink strength (Wei et al., 2019). Furthermore, this study also found that IAA content in inferior grains was consistently lower than that in superior grains, ABA was lower in the early filling stage but higher in the late stage, and GA content remained relatively high throughout. This hormonal profile is unfavorable for sink construction in the early filling stage and for starch accumulation in the mid–to–late stages, representing a key reason for the delayed development and low starch accumulation efficiency in inferior grains.

## 5 Conclusions

In this study, the N180 treatment significantly enhanced leaf antioxidant enzyme activities, optimized the balance of endogenous hormones, and improved post–anthesis photosynthetic performance, thereby ensuring a sustained supply of photoassimilates to the grains. Concurrently, the N180 treatment optimized the endogenous hormone balance in both superior and inferior grains, increased the activities of AGPase and SSS, and strengthened the starch accumulation capacity of the grains. Additionally, throughout the grain–filling period, the contents of IAA, GA, and ABA consistently differed significantly between superior and inferior grains. During the mid–to late–filling stages, the ABA and GA levels in inferior grains were higher than those in superior grains, which became a major factor constraining sink establishment and starch accumulation. Moreover, the insufficient activity of key starch–synthesizing enzymes during the mid–filling stage further exacerbated the sink–activity limitation in inferior grains. In summary, under the experimental conditions of this study, the N180 treatment coordinately enhanced the photosynthetic performance of source leaves and the sink activity of grains, promoted the synchronized development of superior and inferior grains, ultimately increased grain starch accumulation and sink–filling capacity, and provided a sound physiological basis for high–yield and high–efficiency cultivation of spring maize.

## Data Availability

The original contributions presented in the study are included in the article/supplementary material. Further inquiries can be directed to the corresponding author.
